# Standardization and quantitative analysis of *Araucaria Heterophylla* extract via an UPLC-MS/MS method and its formulation as an antibacterial phytonanoemulsion gel

**DOI:** 10.1038/s41598-022-16188-1

**Published:** 2022-07-22

**Authors:** Nermin A. Younis, A. Hemdan, Mai M. Zafer, Wessam H. Abd-Elsalam, Samar M. Abouelatta

**Affiliations:** 1 Department of Pharmacognosy, Faculty of Pharmacy, Ahram Candian University, 6 October, Cairo, Egypt; 2 Department of Pharmaceutical Analytical Chemistry, Faculty of Pharmacy, Ahram Canadian University, 6 October, Cairo, Egypt; 3 Department of Microbiology and Immunology, Faculty of Pharmacy, Ahram Candian University, 6 October, Cairo, Egypt; 4 Department of Pharmaceutics and Industrial Pharmacy, Faculty of Pharmacy, Cairo University, Kasr El-Ainy Street, Cairo, 11562 Egypt; 5 Department of Pharmaceutics, Faculty of Pharmacy, Ahram Candian University, 6 October, Cairo, Egypt

**Keywords:** Microbiology, Plant sciences, Nanoscience and technology

## Abstract

Skin infections are among the bacterial infections that present significant therapeutic challenges due to antibiotic resistance. Recently, herbal products clutched a significant attention as safe replacements for other medications but their low aqueous solubility and poor bioavailability are considered major challenges which could be circumvented via formulation. As a species of genera *Araucaria*, *Araucaria Heterophylla* possesses pharmacological activities such as antioxidant and antibacterial actions, and this study aimed to standardize the extract of the plant against 4ʹʹʹmethoxyamentoflavone (as a main component of the extract) through a validated UPLC-MS/MS method and evaluate its antibacterial activity, which was followed by loading the standardized extract into a nanoemulsion to form a phytonanoemulsion (PNE), where the design analysis and optimization were performed through a simplex lattice design. The optimized PNE (PNE 3) was then loaded into HPMC/Pluronic F-127 gel (in ratio 1:4) to sustain the release of the active constituent. The heightened penetrability of PNE 3 gel was visualized via confocal laser scanning microscopy, and its prolonged effect was proved thru an in vivo study conducted on male Wistar rats. A histopathological study revealed the safety of the formulation when applied topically. Thus, PNE gel could be a potentially broad-spectrum antibacterial drug delivery system.

## Introduction

As the largest organ of the human body, the skin acts as a natural obstacle to prevent pathogenic invasions and if it is broken or the equilibrium between commensals and pathogens is agitated, skin disease or even systemic disease can result^1^. Skin and soft tissue infections (SSTIs) contribute greatly to infections in the hospital population and they are higher among the common bacterial infections, thus posing significant therapeutic challenges. In healthcare settings, the most frequent bacterial contaminants are *Staphylococcus spp., Staphylococcus aureus Escherichia coli, Pseudomonas aeruginosa, and Micrococcus luteus,* which results in hospital-acquired infections, particularly in immunocompromised patients^2^. In particular, *Staphylococcus aureus* and *Escherichia coli* are among the main reasons of hospital-associated SSTIs, that range from superficial abscesses to life-threatening illnesses^3^. In this situation, the strategy adopted for the management of skin bacterial infections is the use of antibiotics whose effectiveness and their selectively toxic nature show penalties that affect their administration. Thoroughly, the administration of antibiotics is associated with negative inferences as to the risk of consequent infection with a resistant microorganism and other agent-specific adverse effects which calls for alternative therapies^4^. Concomitantly, plant-derived compounds have a long history of treating microbial infections^5^, and recently herbal medicine has been utilized as a replacement for traditional medications^6^. Therefore, new trends in developing advanced herbal-based products with lower side effects have evolved^7^.

Family Araucariaceae is an ancient family of coniferous trees and contains four genera including; *Agathis Salisb., Araucaria Juss., Columbea Salisb.*, and *Wollemia* W.G.Jones, K.D.Hill and J.M.Allen^8^. Different species of the genera *Agathis*, including; *Agathis dammara*, *Agathis alba, Agathis robusta, Agathis Araucana*, and *Agathis columnaris,* have shown an antibacterial activity against *Staphylococcus aureus, Micrococcus luteus, Escherichia coli, Pseudomonas aeruginosa,* and other bacterial species^8^*.* Moreover, some of the species of genera *Araucaria* which are represented by 19 species^9^, such as *Araucaria angustifolia* has revealed an antibacterial activity against Gram-negative and Gram-positive strains^10^. The medicinal significance of the genus *Araucaria* (Family- Araucariaceae) is primarily accredited to its different phytochemical constituents, including; Biflavanoid, diterpene, phenylpropanoid, and lignans. The pharmacological activity of Genus *Araucaria* in modern medicines includes anti-inflammatory, antiulcer, antiviral, neuroprotective, antidepressant, and anticoagulant effects^11^. As a species of genera *Araucaria*, *Araucaria Heterophylla* (*A. Heterophylla*) possesses many pharmacological activities such as antiulcerogenic activity^12^, antibacterial activity^13^, antioxidant, anticancer activities^14^, and toxoplasmicidal activity^15^.

Over the past decade, the formulation of nanosystems containing either the extract or the phytoconstituents of medicinal drugs such as *A. Heterophylla* has been adopted as a new approach to increase the medicinal potentialities, including; maximizing the stability of the active components, minimizing the toxicity, decreasing the volatility, and enhancing the penetration of phytochemicals in the tissues and thus improving their cellular uptake^16^. This is because poor aqueous solubility and large molecular weight of phytoconstituents result in low absorption due to the difficulty to cross lipid membrane, and consequently, low bioavailability and low efficacy^17^. Accordingly, nanotechnology was adopted to encapsulate herbal drugs into drug delivery systems thus improving their solubility, minimizing dose, and enhancing active absorption of drugs leading to an elevated bioavailability of herbal medicines and thus increased patient compliance^18,19^. In particular, new nanoformulations were evolved for treating skin and soft tissue infections and promoting wound healing, including nanogels^20^, hydrogel sheets^21^, and nanoVelcro multifunctional wound dressing^22^.

In this context, Nanoemulsion (NE) is considered a useful approach for the delivery of therapeutic molecules^23^. NEs are spontaneously-forming transparent systems with droplets in the range of 10–100 nm, where oils and water are emulsified with a surfactant or a mixture of surfactants, usually in combination with a co-surfactant, in optimal ratios. NEs are versatile systems; either oil-in-water or water-in-oil and they are considered thermodynamically stable as per the interfacial film composed of conjugated surfactant and cosurfactant molecules^24^. NEs were demonstrated to have the ability to solubilize a wide range of compounds^25^, in addition to their easy and cheap scale-up for industrial production, where no specialized equipment is required^26^. Moreover, NEs have been exploited as drug delivery systems for a wide range of pharmaceutical compounds via different routes; including, topical, transdermal, oral, and ocular^27^. As topical formulations, NEs promote the drug penetration into the tissue layers, improving its efficacy^28^. The surfactants in NEs serve as penetration enhancers, that disrupt the skin barrier functions of the stratum corneum which acts as a lipotropic barrier that confines the penetration of molecules^29^. In addition, NEs possess nano-sized droplets which can penetrate through deeper skin layers effortlessly and use the skin as a depot for sustained drug release; thus they are convenient delivery systems for topical application^30^. Owing to their low viscosity, NEs are intended to be incorporated into gel bases, to increase their retention at the site of application, guaranteeing enhanced drug absorption, and hence better therapeutic outcomes.

Therefore, the objective of this work was to conduct a comprehensive study including the extraction and the standardization of the methanolic extract of *A. Heterophylla* via a validated UPLC-MS/MS method, as well as, assessing its antimicrobial activity. Following that, an approach to develop a suitable topical phytonanoemulsion (PNE) gel system to enhance the biological activity of the standardized extract of *A. Heterophylla* (SAH) was attempted through a simplex lattice design using Design expert^®^ software to optimize the PNE systems, and thereafter, loading the optimized PNE into a gel base. To test the enhanced penetration potentiality of the developed formulation, the penetration of the optimized PNE gel was investigated via a confocal laser scanning microscopy study. In addition, the deposition of SAH into the rat’s skin; following the topical application of SAH, the optimized PNE, and the optimized PNE gel, was assessed. Furthermore, a histopathological study was conducted on rats to validate the biosafety of the optimized PNE gel.

## Materials and methods

### Materials

#### Plant material

Samples of non-flowering aerial parts of cultivated *A. Heterophylla* were collected from Prince Mohamed Ali's palace garden (Cairo, Egypt) in August 2019, where the collection procedure was in compliance with the national and international guidelines and legislation. The identification of the plant material was confirmed by Dr. Therese Labib (senior head of specialists in plant identification). A voucher specimen (AH-6 *A. heterophylla*) was deposited in the herbarium of the Pharmacognosy Department, Faculty of Pharmacy, Ahram Canadian University.

#### Analytical materials and apparatus

Silica gel G60 (E-Merck, Darmstadt, Germany) for Column chromatography and Sephadex LH-20 (GE Healthcare Bio-Sciences AB Uppsala, Sweden) were used. Analytical precoated TLC silica gel 60 GF _254_ and Preparative TLC silica gel G (E-Merck, Darmstadt, Germany) were employed in TLC examination. LC/MS**-**grade Acetonitrile, Ethanol, formic acid (Sigma-Aldrich, Germany), and deionized water were used all over the work. ^1^H and ^13^C NMR were recorded using a Varian Mercury instrument (1H-, 500MHZ, 13C-, 75 MHz), and Tetramethylsilane (TMS) was included as an internal standard. UPLC-MS/MS method was adopted for the separation using a Shimadzu LC-2040C, 3D PLUS Nexera-i series LC system coupled with a triple quadrupole 8040 mass spectrometer, LC-2040 pump, and 4-line degasser (Kyoto, Japan). The separation was achieved on Shim-pack GIST-HP C18 (150 mmL.x3.0 mmI.D., 3 μm) analytical column (Kyoto, Japan), and data acquisition was performed using LabSolutions software (Shimadzu, Kyoto, Japan).

#### Test organisms

Two pathogenic bacterial strains; a Gram-positive *Staphylococcus aureus* (ATCC 6358) and a Gram-negative *Escherichia coli (*ATCC 25923*) in addition to the fungus Candida albicans (*ATCC 10231) were used as test organisms*.* Bacterial cultures were sub-cultured on Mueller Hinton agar (MERCK; Germany) and the fungus was sub-cultured on potato dextrose agar (MERCK; Germany).

#### Materials for formulation

Maisine and Capryol 90 were acquired from Gattefossé (St-Priest, France). Cremophor EL and Pluronic F127 were procured from BASF Co. ((Florham Park, New Jersey, USA). Transcutol and hydroxypropyl methylcellulose (HPMC,15000 cp) were bought from Sigma Aldrich Chemical Co. (St. Louis, MA). Phosphate salts (potassium and sodium) were purchased from Merck (Darmstadt, Germany).

### Methods

All methods were carried out in accordance with relevant guidelines and regulations.

#### Extraction and isolation of 4ʹʹʹMethoxyamentoflavone

The dried aerial parts of *A. Heterophylla* (1 kg) were finely powdered and extracted with methanol and then left at 25 ± 2 $^\circ$C with frequent agitation. The procedure was performed two times and the combined extract was evaporated under vacuum at 40 °C to form a green residue (135 g). A part of the residue (80 g) was analyzed via silica gel column chromatography. The elution was conducted using dichloromethane (CH_2_Cl_2_) and then with dichloromethane-methanol (CH_2_Cl_2_-MeOH) in the direction of increasing polarity up to 20% MeOH in CH_2_Cl_2_. The process resulted in the separation of 50 portions (100 mL each). The collected portions were scrutinized by TLC using the solvent system CH_2_Cl_2_-MeOH (7:1), examined under UV, then followed by spraying with either ferric chloride (FeCl_3_) or 20% sulphuric acid in ethanol. All portions eluted with (92:8) CH_2_Cl_2_-MeOH were alike, so they were added to one another, and upon TLC analysis they were found to comprise a compound (**A**). The combined portions were repetitively chromatographed on silica gel TLC plates and eluted with CH_2_Cl_2_-MeOH (7:1). Repeated purification on Sephadex LH-20 column eluted with MeOH, afforded compound (**A**) (70 mg). The physicochemical properties of 4ʹʹʹmethoxyamentoflavone were determined using the Swiss ADME tool.

#### UPLC-MS/MS standardization of *A. Heterophylla*

##### Preparation of standard solutions and working solutions

A primary stock solution of standard 4ʹʹʹMethoxyamentoflavone of concentration 5.0 mg/mL was prepared by dissolving 50 mg of the standard powder in an ethanol/deionized water mixture in a 10 mL volumetric flask, which was then filtered via a millipore filter (0.2 µm). The primary stock solution was diluted with deionized water to prepare standard working solutions (3 mg/mL). Ten milligrams of the dried extract were reconstituted in 10 mL ethanol (70% v/v) and sonicated till completely dissolved. It was made up to the final volume (100 mL) with ethanol (70% v/v), then filtered via a millipore filter (0.2 µm), where this solution was employed for the standardization of the extract.

##### UPLC-MS/MS analysis

The extract and the standard compound were analyzed by UPLC connected to triple quadruple 8040 MS. The used UPLC column was Shim-pack GIST-HP C18 (150 mmL. × 3.0 mmI.D., 3 μm). Gradient elution was performed where the employed mobile phases were: phase A: 0.1% formic acid in acetonitrile; phase B: 0.1% formic acid. The mobile phase gradient was set as follows: 90% B from 0 to 1 min at a flow rate of 0.5 mL/min; then linearly decreased to 60% B from 1 to 2.5 min; this was followed by decreasing to 30% B while the flow rate was increased to 1 mL/min from 2.5 to 3.5 min, maintained at 30% B and flow rate of 1 mL/min from 3.5 to 4.5 min, then increased to 90% B at a flow rate of 0.5 mL/min from 4.5 to 5.5 min, which was kept maintained from 5.5 to 7 min. The analysis was operated at the selected ion monitoring (SIM) mode. The detection was accomplished on the triple quadrupole mass spectrometer equipped with an electrospray ionization mass (ESI) interface at – 3.5 kV in a negative ionization mode. Desolvation line (DL) temperature and heat block temperatures of 250 $^\circ$C and 400 $^\circ$C were applied, respectively. The flow rates of nebulizing gas and drying gas were set at 3 L/min and 15 L/min, respectively. The analytical run time was 7 min and the full scan covered the mass range from 100 to 1000 m/z.

##### Method validation

For the determination of the method linearity, different concentrations of standard 4ʹʹʹMethoxyamentoflavone and SAH were accurately prepared in the ranges 100–2000 μg/mL and 10–400 μg/mL, respectively. Following this, a volume of 10 μL of each concentration was injected thrice into the UPLC-MS/MS system. The linear regression model was applied to gain the calibration curve equations and determination coefficients, where the method is considered linear over the selected working range if the determination coefficient (r^2^) value of 0.992 or greater. To check the accuracy of the method, three replicates of different concentrations of the analyte were analyzed. The concentrations were attained from the regression equation, and the percentage recoveries were calculated. According to ICH guidelines, the precision (repeatability) should be assessed based on a minimum of 9 determinations covering the reportable range for the procedure (e.g., 3 concentrations/3 replicates each)^31,32^. Consequently, the precision of the method was checked by applying the proposed method to determine three concentrations (15, 40, and 200 µg/mL) of the analyte thrice intra-daily and then calculating the relative standard deviations (RSD). In addition, the process was repeated inter-daily (intermediate precision) on three different days for the three selected concentrations thrice, and RSD was calculated. Determination of the limit of quantitation (LOQ) and limit of detection (LOD) can be accomplished through several approaches as per ICH recommendations. In this context, the determination of the signal-to-noise ratio is achieved by linking measured signals from samples with known low concentrations of analyte with those of blank samples and establishing the minimum concentration at which the analyte can be reliably detected. A signal-to-noise ratio between 3:1 is mostly considered acceptable for estimating the detection limit whereas a ratio of 10:1 is used to estimate the quantitation limit.

#### In vitro antimicrobial and antifungal assays of the standardized *A. Heterophylla* extract:

##### Antibacterial activity

The antibacterial activity of SAH was accomplished using Agar well diffusion method as previously described by Das et al*.*^33^. Culture plates were allowed to solidify and then a volume of 100 μL (10^6^ CFU/mL) of fresh microbial cultures of *Staphylococcus aureus* and *Escherichia coli* was streaked onto Mueller Hinton agar (MHA) for the assay of bacterial susceptibility. The plates were then punched with a sterile cork borer and these opened wells were inoculated with 10 µL of SAH at a certain concentration. Standard antibiotic doxycycline and diluted DMSO were applied as positive and negative controls, respectively. All plates were incubated at 37 °C for 24 h. The experiment was conducted in triplicate and the average zone size was calculated. The determination of minimum inhibitory concentration (MIC) was performed in the 96-well microtiter plate according to the micro-broth dilution method reported by Klancnik et al*.*^34^. A concentration of 20 mg/mL of SAH was prepared and 200 μL was added to the first well, then a 100 μL of SAH was subjected to two-fold serial dilution in each well to obtain concentrations of 10, 5, 2.5, 1.25, 0.625, 0.312, 0.156, 0.078, 0.039, 0.0195, and 0.00975 mg/mL, respectively, in Mueller Hinton broth. The preparation of the inoculum was performed by fine-tuning the turbidity from an overnight microbial culture to a value equivalent to McFarland 0.5 which was then diluted to a final concentration of 10^6^ CFU/mL. 100 μL of the inoculum was mixed with comparable volumes of the two-fold serially diluted SAH in a microplate. Culture medium, bacterial suspension, SAH, and DMSO in amounts corresponding to the highest quantity presented were set as controls. The incubation was performed for 24 h at 37 °C and the MIC was established as the lowest concentration showed no visible growth. The mean value of three replicates was calculated.

##### Antifungal activity

The agar well diffusion method was employed to examine the antifungal activity of SAH^35^. In brief, *C. Albicans* ATCC 10,231 was accustomed spectrophotometrically at 530 nm so that a final concentration matching the 0.5 McFarland standard was attained. The produced inhibition zones were determined after incubation of the plates at 28 °C for 24 h. Standard antibiotic amphotericin and diluted DMSO were used as positive and negative controls, respectively. All procedures were performed thrice, and mean values were calculated. To determine the minimum fungicidal concentration which is defined as the lowest concentration showing no growth on potato dextrose agar after 48 h of incubation, serial two-fold dilutions of SAH were prepared with potato dextrose agar to obtain final concentrations ranging from 30 to 1.6 mg/mL. Aliquots were allocated in 96-well microplates. Inoculum of *C. Albicans* was prepared to a final concentration of 2 × 10^3^ CFU/mL in potato dextrose agar medium and then 100 μL of the suspension was added to each well. In the case of wells showing no growth, a 100 μL was transferred on potato dextrose agar plates and incubated for 48 h.

#### Formulation of phytonanoemulsion gel system containing the standardized *A. Heterophylla* extract

##### Construction of pseudo-ternary phase diagram

The preparation of NEs was accomplished using the phase titration method^36^ at 25 °C. The type and ratio of oils and surfactants were selected according to preliminary studies (Data not shown). In brief, oils (Maisine and Capryol 90, in ratio 1:1), and surfactant/cosurfactant mixtures (S_mix_) (Cremophore EL/Transcutol, in ratio 2:1) were blended at different ranges; from 9:1 to 1:9 (w/w), then vortexed at 1500 rpm for 3 min. The oils and S_mix_ blends were then titrated with distilled water; drop by drop, and the endpoint of the titration was the point of turbidity of the mixture, at which the amount of the aqueous phase was recorded^37,38^. The corresponding pseudo-ternary phase diagram was plotted via Tri-plot software (Ver. 4.1.2, Leicestershire, England), and the transparent NEs region was contoured.

##### Simplex Lattice Design

The optimization of the systems was achieved through a simplex lattice design employing Design expert^®^ software (Version 7, Stat-Ease Inc., MN, USA). The components of the NE (The oil (A), the water (B), and the S_mix_ (C)) were defined as the independent variables. Based on the region which corresponds to the clear systems in the pseudo-ternary phase diagram, the higher and lesser percentages of formulating constituents were set as follows:

1 $${5 } \le {\text{ A }} \le { 25 }\left( \% \right)$$

2 $${25 } \le {\text{ B }} \le { 45 }\left( \% \right)$$

3 $${5}0 \, \le {\text{ C }} \le { 7}0 \, \left( \% \right)$$

4 $${\text{A}} + {\text{B}} + {\text{C}} = { 1}00 \, \left( \% \right)$$

The dependent variables were designated as follows; droplet size (DS)(Y_1_), polydispersity index (PDI)(Y_2_), percentage transmittance (%T) (Y_3_), and the cumulative percent of drug released after 8 h (Q_8_) (Y_4_). In consideration of small values of DS and PDI and large values of %T and Q_8_, the optimized NE would be recommended based on the desirability function. The phytonanoemulsions (PNEs) were prepared by loading the NEs with SAH at 20% (w/w), and the composition of the design-proposed PNEs is recorded in Table 1.

**Table 1:** Composition of the prepared PNEs and their observed responses.

Formulations	A	B	C	Y_1_	Y_2_	Y_3_	Y_4_
	Oil %	Water %	S_mix_ %	DS* (nm)	PDI*	%Transmittance*	Q_8_*
PNE 1	5.00	25.00	70.00	32.50 ± 2.83	0.17 ± 0.00	96.60 ± 0.71	97.33 ± 1.41
PNE 2	5.00	35.00	60.00	72.29 ± 6.08	0.35 ± 0.01	93.50 ± 1.34	99.08 ± 9.95
PNE 3	5.00	45.00	50.00	44.93 ± 0.60	0.17 ± 0.04	94.30 ± 0.00	97.56 ± 1.51
PNE 4	8.33	28.33	63.33	86.54 ± 3.94	0.29 ± 0.03	96.30 ± 2.25	81.49 ± 4.36
PNE 5	8.33	38.33	53.33	89.10 ± 9.90	0.35 ± 0.01	93.70 ± 3.46	90.52 ± 1.79
PNE 6	11.67	31.67	56.67	93.47 ± 2.21	0.34 ± 0.03	85.40 ± 0.95	88.60 ± 5.21
PNE 7	15.00	25.00	60.00	86.53 ± 7.05	0.38 ± 0.06	84.50 ± 0.07	87.84 ± 6.84
PNE 8	15.00	35.00	50.00	99.87 ± 3.93	0.48 ± 0.07	80.85 ± 2.61	85.19 ± 1.25
PNE 9	18.33	28.33	53.33	120.55 ± 1.12	0.43 ± 0.03	77.80 ± 1.66	91.90 ± 5.54
PNE 10	25.00	25.00	50.00	127.65 ± 4.38	0.53 ± 0.05	76.15 ± 3.50	72.07 ± 2.45

* Mean ± Standard deviation (n = 3).

Abbreviations: PNE, Phytonanoemulsion; S_mix_, Surfactant/Cosurfactant mixture; DS, Droplet size; PDI, Polydispersity index; Q_8_, The cumulative percent of SAH released in 8 h.

##### In vitro characterization of PNEs

*Droplet size (DS), Polydispersity index (PDI), and Percentage Transmittance (%T) measurements.* The average DS and PDI of the prepared PNEs were assessed via the dynamic light scattering technique (Zetasizer Nano ZS, Malvern Instruments, Worcestershire, UK). %T of the freshly prepared PNEs was measured at 550 nm via a UV–visible spectrophotometer (Shimadzu, Kyoto, Japan). All measurements were carried out in triplicates and recorded as average ± standard deviation.

*In vitro dissolution test.* The test was executed via the dialysis membrane method in an incubator shaker (Unimax, IKA, Germany). Samples (0.5 g) of SAH and PNEs (containing SAH equivalent to 100 mg) were packed in cellulose dialysis membranes which were sealed tightly from both sides and then immersed in bottles filled with 50 mL phosphate buffer (pH 6) containing 10% ethanol to maintain the sink condition. The incubator shaker was operated at 60 strokes per minute and the temperature was maintained at 32 ± 2 $^\circ$C. Aliquots (2 mL) were withdrawn from each bottle at specified time intervals and replenished immediately with a fresh medium. The content of SAH in the samples was analyzed via UPLC-MS/MS method as stated previously under “UPLC-MS/MS analysis” section.

*Transmission electron microscopy (TEM).* The morphology of the optimized PNE was observed using transmission electron microscopy (Joel JEM 1230, Tokyo, Japan). A volume of 50 µL of PNE was sited on a metallic grid and the surplus was removed with a filter paper, and the grid was then left to dry in the air. Finally, the grid was visualized and photographed via TEM^39^.

##### Preparation of the optimized PNE gel

In general, the viscosity of NE systems is practically low, and to increase the formulation retention at the affected parts, the viscosity of the systems has to be elevated through its incorporation in a gel base. In this study, the plain gel base was prepared by mixing hydroxypropyl methylcellulose (HPMC) and pluronic F127 at a ratio of 1:4 (w/w) on a magnetic stirrer for 30 min at room temperature, then the optimized PNE (1 g) was added to the gel base (1 g) and mixed on a magnetic stirrer for 10 min at room temperature.

##### Characterization of the optimized PNE gel

*pH and rheological measurements.* The apparent pH of the optimized PNE gel was determined via a pH meter (Jenway, UK). The viscosity of the optimized PNE gel was determined using a Brookfield viscometer (DV-II Programmable Rheometer, Brookfield Engineering LABS, Stoughton, MA) fitted with a cone spindle 40. The spreadability was examined as per the procedure stated previously by Abd-Elsalam et al*.*^40^, where 1 g of the preparation was loaded in between two glass slides, and a 200 g weight was situated on the upper glass slide for 1 min to uniform the thickness. Consequently, the area up to which the formulation was able to spread (spreadability) was measured and the spreadability was calculated via the equation:

5 $${\text{S }} = \, \left( {{\text{M}}.{\text{A}}} \right)/{\text{T}}$$

where S = spreadability (g.cm. sec^-1^), M = weight (200 g), A = area of formulation spread on the slide and T = time (60 s).

#### In vitro dissolution test and accelerated stability studies

The in vitro dissolution test of the optimized PNE gel was completed as formerly declared under “In vitro dissolution test” section. To evaluate the system stability, the optimized PNE gel was stored at accelerated storage conditions (40 °C ± 2 °C/75% RH ± 5% RH) for 3 months as per ICH guidelines. The stored formulation was periodically evaluated for any change in appearance, pH, or viscosity^26^.

#### In vivo studies

##### Confocal laser scanning microscopy (CLSM) study

The protocol for handling the laboratory animals was approved by the Research Ethics Committee, Faculty of Pharmacy, Cairo University, Egypt (PI 2970). All employed procedures followed the recommendations in the ARRIVE guidelines. Nine male Wistar rats were separated into three groups, with three animals each, and the hair in the dorsal area was depilated and a specific area was circumscribed. Group (A), Group (B), and Group (C) were treated topically with a specific volume of Rhodamine B dye solution, Rhodamine B dye-loaded PNE, and Rhodamine B dye-loaded PNE gel (each containing 5 mg Rhodamine B dye) onto the circumscribed area, respectively. After 8 h, the rats were decapitated, and the dorsal skin was separated, rinsed with saline solution, and finally sliced by a microtome (SM2400; Leica Microsystems, Wetzlar, Germany). CLSM was employed to inspect the dermal distribution of Rhodamine B dye^41^.

##### Skin deposition study

Sixty-three male Wistar rats were distributed into 3 groups, 21 animals in each. Group A, B, and C received topically SAH, the optimized PNE, and the optimized PNE gel, respectively. At the beginning of the experiment, the depilated dorsal skin was circumscribed and 0.5 g of the tested samples (containing the equivalent of 100 mg SAH) were rubbed on the marked area. At sampling points (1, 2, 3, 4, 6, 8, and 24 h), 3 animals were selected from each group and then decapitated and their dorsal skin was removed and rinsed with saline solution. The obtained skin samples were shredded into small pieces, then drenched into 5 mL methanol and homogenized then filtered through a 0.45 mm membrane filter^41^. Aliquots of the supernatants were withdrawn and assayed via the UPLC-MS/MS method mentioned earlier under “UPLC-MS/MS analysis” section.

##### Statistical analysis

Data were represented as mean ± S.D. (n = 3). The area under the curve (AUC_0-24_) in the plot of the amount of SAH deposited (µg/cm^2^) versus time (h) was calculated via the PK solver integrated into Microsoft Excel, which gives an idea about the degree of SAH dermal deposition upon the administration of the tested preparations. The differences in the values of AUC_0-24_ were statistically checked by one-way ANOVA, tailed by Tukey’s HSD (Honest Significant Difference) test, where the difference was established to be significant at a *P* value less than 0.05.

##### Histopathological study

The experiment was designed so that six animals were divided into two equal groups. The hair in the dorsal area was depilated and a specific area was circumscribed. Group (A) served as a control group (received no treatment), while Group (B) was topically treated with 0.5 g of the optimized PNE gel (containing the equivalent of 100 mg SAH) once daily for a whole week. The rats’ dorsal skins were observed for any sign of irritation; including, erythema and edema. Finally, the animals were decapitated and the dorsal skin was separated, cleaned with saline solution, and maintained in 10% formalin^42^. The skin specimens were then dehydrated with alcohol gradient, and immersed in paraffin wax, which was then allowed to harden to form blocks^43^. Rotatory microtome was used to slice sections of tissue (4 µm) which were then loaded on glass slides and finally tainted by Hematoxylin and Eosin. The tissue samples were then visualized with a light microscope and photographed simultaneously.

## Results and discussion

### Identification of 4ʹʹʹmethoxyamentoflavone by ^1^H NMR and ^13^ C

**Chemistry: **^1^H and ^13^C NMR data of compounds **A** is shown in Table 2**.** The elucidation and identification of the compound structure were performed via different spectroscopic analyses and the results were compared to the published literature. The compound was identified as 4'" methoxy amentoflavone.

**Table 2:** ^1^H NMR and ^13^C NMR spectrum of compound A in acetone-d_6._

No	^13^C NMR	^1^H NMR	No	^13^C NMR	^1^H NMR
**2**	165.4		2"	165.5	7.6 (d, J = 8 Hz)
**3**	101.8	6.54 (s)	3"	102.0	6.34 (s)
**4**	182.1		4"	182.2	
**5**	162.0		5"	160.0	
**6**	98.7	6.04 (d, J = 2.0 Hz)	6"	101.8	6.31 (s)
**7**	165.02		7"	162.1	
**8**	94.0	6.1 (d, J = 2.0 Hz)	8"	102.2	
**9**	158.0		9"	155.5	
**10**	102.2		10"	108.0	
**1'**	120.0		1'"	123.9	
**2'**	131.6	8.2 (d, J = 2.0 Hz)	2'"	127.9	7.67(d, J = 8.8 Hz)
**3'**	123.9		3'"	114.0	6.44 (d, J = 8.8 Hz)
**4'**	158.0		4"'	162.1	
**5'**	120.0	7.04 (d, J = 8.7 Hz)	5'"	114.0	6.44 (d, J = 8.8 Hz)
**6'**	130.5	7.80 (dd, J = 2, 8.7 Hz)	6'"	127.86	7.67 (d, J = 8.8 Hz)
			4"'OCH3	54.7	3.65(s)

**Figure 1:**
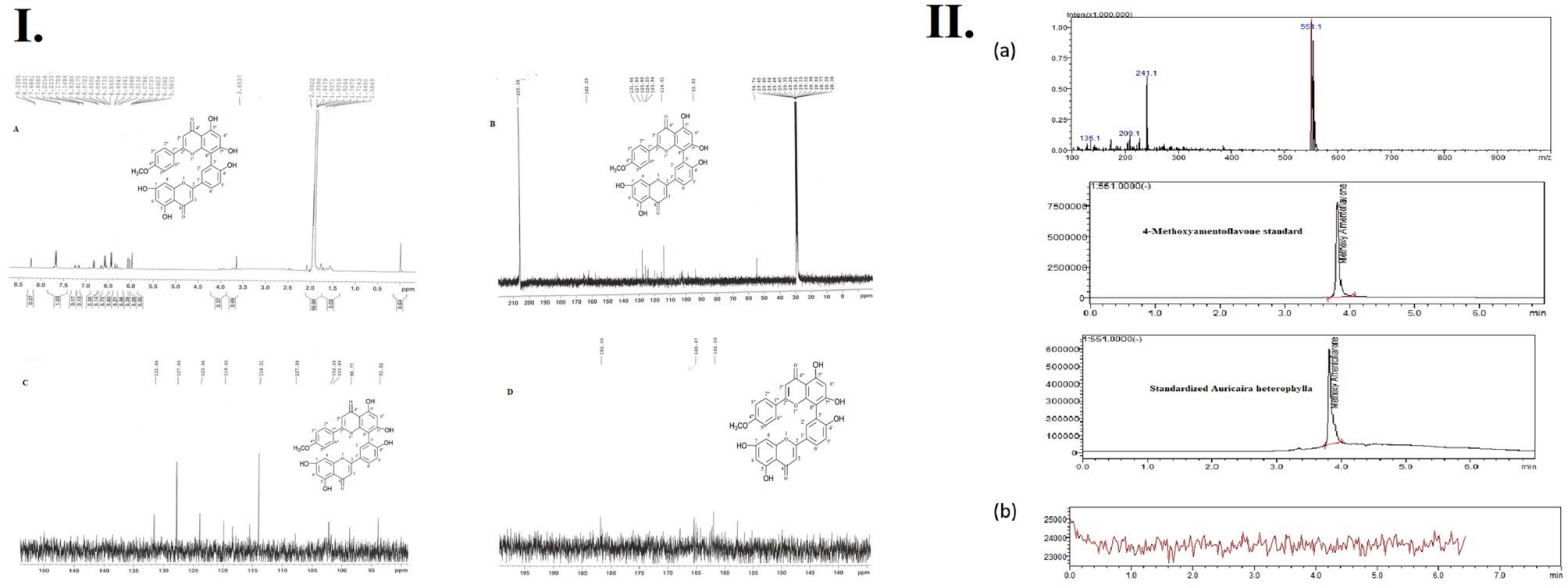
(**I**) Compound **A** ^1^H NMR and ^13^ C NMR data, and (**II**) (**a**) Selected Ion Chromatograms, retention times, and full scan mass spectra (top), Selected Ion Chromatogram and retention time of 4ʹʹʹmethoxyamentoflavone standard (middle) and Selected Ion Chromatogram and retention time of SAH (bottom), and (**b**) Blank samples appeared free from any interfering substances at the retention time and m/z of the studied compound.

***Compound A*** was obtained as an amorphous yellow powder (70 mg). The compound represented the chief constituent of the methanol extract of *A. Heterophylla* and it was anticipated to be a phenolic compound due to its strong FeCl_3_ reaction. Compound **A** was identified principally through the interpretation of the ^1^H and ^13^C NMR data, Fig. 1I. The ^1^H NMR spectrum showed two meta-coupled proton signals of H-6 and H-8 appeared at δ 6.04 ppm (d, *J* = 2 Hz) and δ 6.1 ppm (d, *J* = 2 Hz), respectively, and signals at δ 7.67 ppm (d, *J* = 8 Hz) and δ 6.44 ppm (d, *J* = 8 Hz) corresponding to the protons H-2″’/6″’ and H-3″’,5″’, which indicated that the compound contains a para-disubstituted aromatic ring. Also, 4'"- methoxy protons showed a singlet signal at δ 3.65 ppm. The ^13^C NMR spectrum showed 31 signals including 4'"- methoxy at δ 54.7 ppm and two carbonyl groups at δ 182.1 and 182.2 ppm. Moreover, It showed a significant downfield shift for C-8`` flavones unit at δ 102.2 ppm; indicating that C-8``was involved in the interflavonoid linkage. Based on the foregoing spectral studies, Compound **A** was characterized as a 3`, 8`` biflavonoid. The existence of two carbonyls along with the analysis of the ^1^H NMR spectrum proposed a flavonoids dimer, containing two conjugated flavone units with stated distinctive peaks for a biflavon nucleus^44^ formerly isolated from *Podocalyx loranthoides* and *Caesalpinia pyramidalis*. Conclusively, the structure of Compound **A** was fully confirmed to be 4ʹʹʹmethoxyamentoflavone (Podocarpusflavona A) by comparing its spectral data to previously published data^45,46^.

### Physicochemical properties of 4ʹʹʹmethoxyamentoflavone as achieved by Swiss ADME

The herbal extract was found to be rich in a biflavonoid known as 4ʹʹʹmethoxyamentoflavone (C_31_H_20_O_10_) having a molecular weight of 552.48 g/mol, and showing poor aqueous solubility (2.26*10^–7^ mg/mL) with a Log P value of 3.75. The identified compound, 4ʹʹʹmethoxyamentoflavone, is known to possess antibacterial activity^47^. Unfortunately, both poor aqueous solubility and large molecular weight of the compound result in low absorption due to the difficulty to cross lipid membrane, which leads to low bioavailability, and thus low efficacy.

### UPLC-MS/MS standardization of *A. Heterophylla* extract and Method validation

Using automatic optimization of LabSolutions software, all the mass conditions were optimized to detect and quantify the studied analyte. The Selected Ion Chromatograms, retention times, and full scan mass spectra are shown in Fig. 1.II.a. Six different non-zero samples were used to assess the linearity of the method, where good linearity was established from 10 µg/mL to 400 µg/mL, with a correlation coefficient of 0.9992 and the regression equation was: y = 260783x − 4.30315e + 006. The accuracy and precision of the method were checked at three concentration levels, where the inter-day RSD was 1.672, while the intra-day RSD was 1.823 as shown in Table 3. The determined LOQ and LOD with acceptable accuracy were 0.50 µg/mL and 0.16 µg/mL, respectively. Moreover, all the studied analytes showed good stability in the whole duration of the study, where their recoveries in all stability determinations ranged from 92 to 103%. It should be noted that the developed method was proved to determine the analyte without interference from other substances that may be extracted in the in vivo studies. This was confirmed by the absence of any interfering substances when the blank skin layer (without application of the medications) of six rats was extracted and analyzed. The blank samples appeared free from any interfering substances at the retention times and m/z of the studied analyte as shown in Fig. 1IIb. After standardization, it was found that 4ʹʹʹmethoxyamentoflavone was the major component, where one gram of SAH contains 20 mg 4ʹʹʹmethoxyamentoflavone. Based on these findings, SAH antibacterial and antifungal activity were evaluated.

**Table 3:** Calibration curve equation and linearity (n = 6), intra-day and inter-day precision, accuracy, and LOQ and LOD.

Range µg/mL	10—400
Regression Equation	y = 260783x − 4.30315e + 006
Correlation coefficient (r)	0.9992
Accuracy^a^	97.24 ± 1.81
Repeatability^b^	98.53 ± 1.796
RSD%	1.823
Intermediate precision^c^	96.28 ± 1.61
RSD%	1.672
LOQ (µg/mL)	0.50
LOD (µg/mL)	0.16

a: Six concentrations covering the range (10–400 µg/mL ).

b: Intra-day (n = 3), an average of three concentrations of the analyte ( 15, 40, and 200 µg/mL ) repeated 3 times within the same day.

c: Inter-day (n = 3), an average of three concentrations of the analyte ( 15, 40, and 200 µg/mL ) repeated 3 times in three consecutive days.

### In vitro antimicrobial and antifungal activities of the standardized *A. Heterophylla* extract:

**Figure 2:**
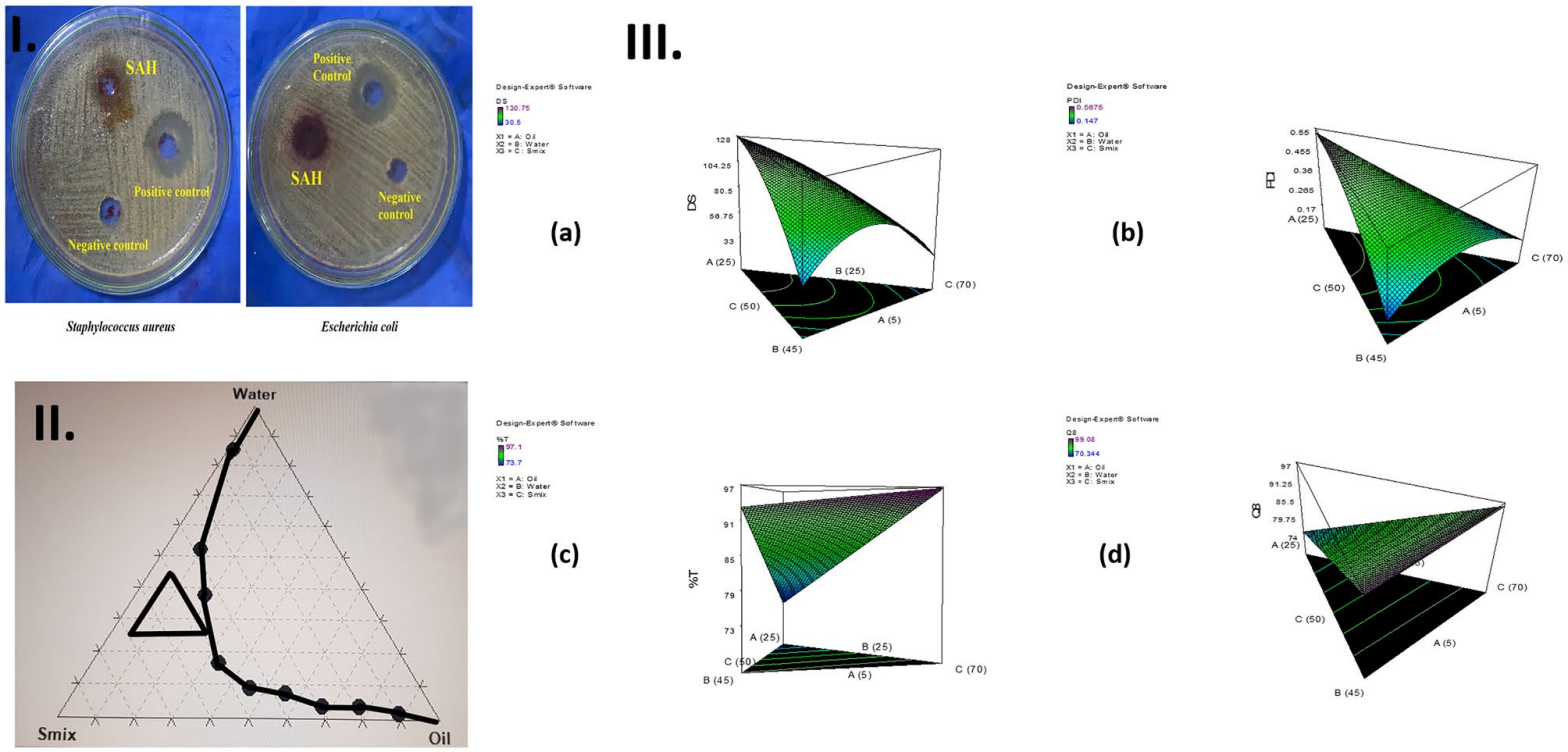
(**I**) Growth inhibition zones of *Staphylococcus aureus* ATCC (6358) and *E. coli* ATCC (25,923) caused by SAH, compared to the positive control (Doxycycline) and the negative control (DMSO), (**II**) Pseudo-ternary phase diagram of oils (Maisine and Capryol 90, in ratio 1:1), Surfactant/CoSurfactant mixtures (Smix) (Cremophore EL/Transcutol, in ratio 2:1), and water. The defined percentages of the oils (5–25%), water (25–45%), and the Smix (50–70%) are represented by the black triangle, **and **(**III**) 3D surface plots demonstrating the effect of the independent variables; the oil (X1), the water (X2), and the S_mix_ (X3) on (**a**) Droplet size in nm (DS), (**b**) Polydispersity index (PDI), (**c**) % Transmittance, and (**d**) The cumulative percent of SAH released in 8 h (Q_8_).

The sensitivity of three standard strains to SAH was examined using the agar well diffusion technique, Fig. 2I. The standard bacterial strains were found to be sensitive to doxycycline while *C.albicans* was found to be sensitive to amphotericin B*. E.coli* showed the highest susceptibility to SAH while SAH showed no effect on *C.albicans* (Table 4). The MIC of SAH was corresponding to 0.156 mg/mL in the case of standard *S. aureus and E.coli,* and more than 30 mg/mL with standard strains of *C.albicans.* The standardized extract showed a significant antibacterial activity which can be attributed to its major bioflavonoids component 4'"methoxy amentoflavone formerly reported to possess a powerful antibacterial activity^48^.

**Table 4:** Antimicrobial activity of SAH.

Standard strain	Zone of inhibition (diameter in mm)		
	SAH	Doxycycline	Amphotericin B
*S. aureus* (ATCC 6358)	8	30	–
*E. coli* (ATCC 25,923)	10	20	–
*C. albicans* (ATCC 10,231)	No effect	–	22

### Construction of pseudo-ternary phase diagram

The choice of oils and S_mix_ was based on the solubility of the herbal extract in different oils, surfactants, and cosurfactants (data not shown). A pseudo-ternary phase diagram was plotted after the preparation of different systems by changing the concentration of the oils, S_mix_, and water, Fig. 2II. The contoured region in the pseudo-ternary phase diagram indicates the nanoemulsion region which helps to choose the optimum concentration of oil, S_mix_, and water. Systems containing more than 30% of the oil phase did not lie within the nano-emulsification region, and S_mix_ concentration lower than 50% resulted in turbid systems, where higher amounts of S_mix_ were required to emulsify the incorporated oils. Consequently and as per the NE area, defined percentages of the oils (5–25%), water (25–45%), and the S_mix_ (50–70%) were selected (represented by the black triangle), which were further included as independent variables in a simplex lattice design.

### Analysis and optimization of the design

A simplex lattice experimental design produced 14 runs (including 4 replicates) generated by Design-Expert^®^ software, the composition of PNEs (loaded with 20% SAH) and the corresponding responses are represented in Table 1. According to the analysis of the obtained data by the software and as per the highest *R*^2^, adjusted *R*^2^, and predicted *R*^2^, and the lowest predicted residual error sum of squares (PRESS), the droplet size (DS)(Y_1_) and polydispersity index (PDI)(Y_2_) followed a quadratic model, while percentage transmittance (%T) (Y_3_) and the cumulative percent of drug released after 8 h (Q_8_) (Y_4_) followed a linear model. Analysis of Variance (ANOVA) was carried out by the software to generate the polynomial equation of the responses. The final equations (in terms of actual components) for the measured responses were as follows;

6 $$\begin{aligned} {\text{DS }} & = \, + 0.{13}*{\text{ Oil }} - {14}.{5}0*{\text{ Water }} - {5}.0{6}*{\text{S}}_{{{\text{mix}}}} \\ & \quad + 0.{16}*{\text{ Oil }}*{\text{ Water }} + 0.{12}*{\text{ Oil }}*{\text{S}}_{{{\text{mix}}}} + 0.{39}*{\text{ Water }}*{\text{S}}_{{{\text{mix}}}} \\ \end{aligned}$$

7 $$\begin{aligned} {\text{PDI }} & = \, + 0.0{19}*{\text{ Oil }} - 0.0{56}*{\text{ Water }} - 0.0{16}*{\text{S}}_{{{\text{mix}}}} \\ & \quad + {1}.0{1}*{1}0^{{ - {3}}} *{\text{ Oil }}*{\text{ Water }} - {1}.{32}*{1}0^{{ - {4}}} *{\text{ Oil }}*{\text{S}}_{{{\text{mix}}}} + {1}.{46}*{1}0^{{ - {3}}} *{\text{ Water }}*{\text{S}}_{{{\text{mix}}}} \\ \end{aligned}$$

8 $$\% {\text{T }} = \, - 0.0{8}*{\text{ Oil }} + 0.{9}0*{\text{ Water }} + {1}.0{7}*{\text{S}}_{{{\text{mix}}}}$$

9 $${\text{Q8 }} = \, - 0.0{7}*{\text{ Oil }} + {1}.0{5}*{\text{ Water }} + 0.{1}0*{\text{S}}_{{{\text{mix}}}}$$

where the sign and the value of the variable coefficient reflect the effect of the studied variables. The positive sign of the variable coefficient designates synergistic effects, and the negative sign designates an antagonistic effect of the factors. Also, it should be noted that the formulation of NE requires an optimum combination of all components, and varying the proportion of any of them would alter the overall equilibrium of the system and thus its measured characteristics.

The influence of the percentage of the components on the measured responses is represented by the 3D surface plot in Fig. 2III. DS and PDI of NEs are considered the most important parameters that keep the drug solubilized, where small DS offers the drug a large surface area and thus increases its opportunity of penetration^49^. In addition, DS plays a crucial role in the drug permeation at the spot of infection and its retaining within the layers of the skin and also affects the stability of NEs^50^. DS of the prepared PNEs ranged from 32.50 ± 2.83 to 127.65 ± 4.38 nm with PDI values in the range of 0.17 ± 0.00–0.53 ± 0.05, which indicated higher uniformity of the globules (Table 1). Regarding the influence of the oil phase percentage on DS and PDI and according to Eqs. (6) and (7), it was clear that scaling up its percentage resulted in increasing DS and PDI due to the expansion of the oily core of PNEs^51^. These findings were consistent with the outcomes previously reported by Shinde et al*.*^52^. A decrease in DS and PDI was observed upon increasing S_mix_, which could be accredited to the solubilization of the internal oily phase within a larger number of surfactant and cosurfactant micelles^53^. These verdicts were comparable to those stated by Dhaval et al*.*^54^. In some formulations, higher percentages of water resulted in the formation of PNEs with smaller DS and lower PDI values due to the dilution effect of water as an external phase of the oil/water PNE, while other formulations showed higher DS and PDI because of varying the proportion of other components. Transparency of PNEs was checked by measuring %T which ranged from 76.15 ± 3.50 to 96.60 ± 0.71%. %T values close to 100% confirmed the nanosize of the droplets^55^. Higher amounts of oil decreased %T values due to the formation of larger droplets. On the contrary, higher percentages of S_mix_ and water resulted in the formation of clearer PNEs having smaller DS and PDI^56^.

**Figure 3:**
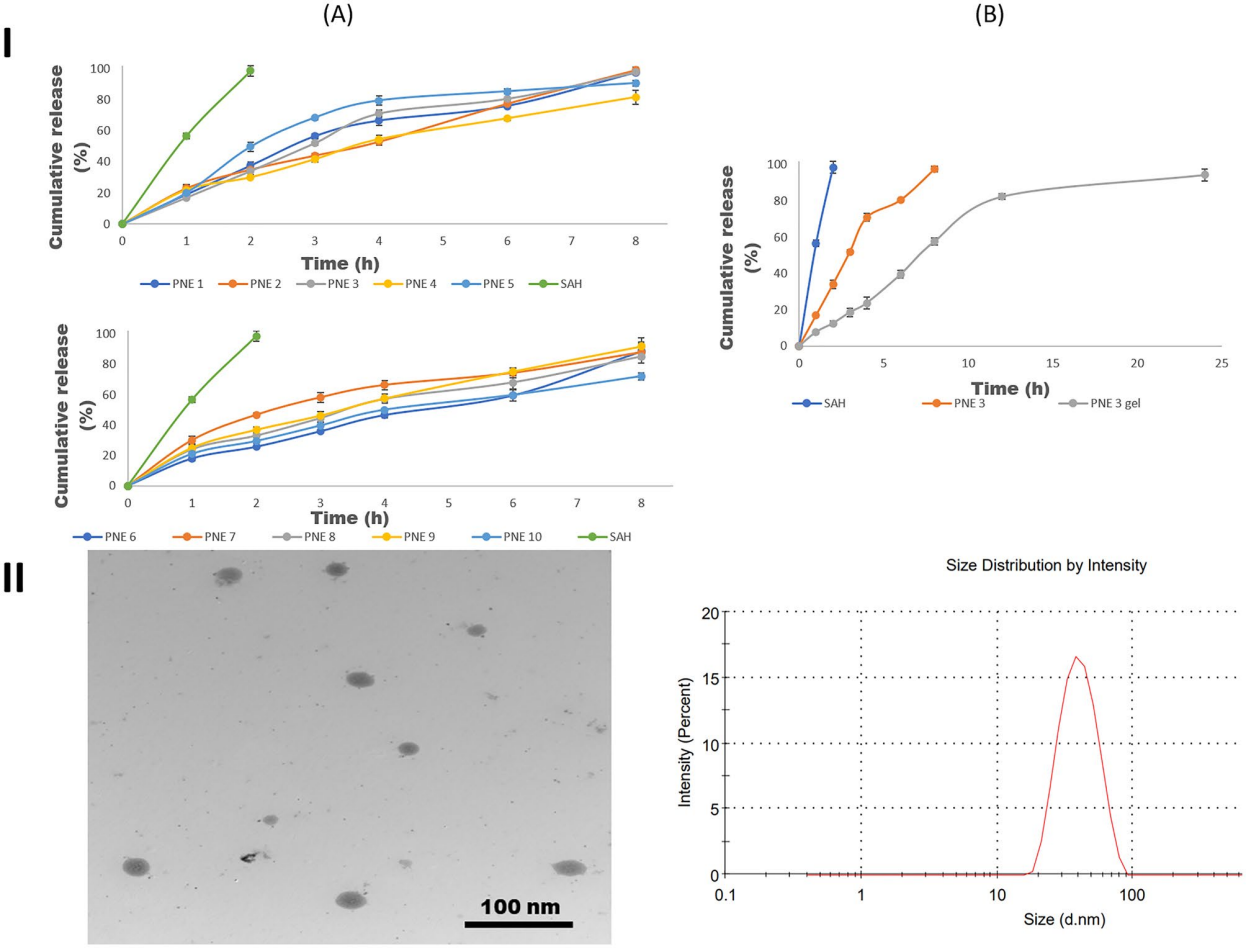
(**I**) (**A**) Release profiles of SAH and PNEs, and (**B**) Release profiles of SAH, the optimized PNE (PNE 3), and PNE 3 gel, in phosphate buffer (pH = 6, simulating skin surface pH) at 32 ± 0.5 $^\circ$C, and (**II**) TEM micrograph of the optimized PNE 3 (Left) and its size distribution graph (Right).

The release profiles of SAH and PNEs are displayed in Fig. 3IA. It could be observed that PNEs were able to sustain SAH release up to 8 h, where the cumulative percent release percentage after 8 h (Q_8_) ranged from 72.07 ± 2.45–99.08 ± 9.95%. Also, it was pragmatic that the degree of the release of SAH from PNEs was slower compared to the extract. The cumulative percent of SAH released after 8 h (Q_8_) was found to be inversely proportional to the size of the droplets^54^. As per these results, the software selected the optimized PNE on basis of minimum DS and PDI, and maximum %T and Q_8_, which coincided with PNE 3 with 0.958 as a desirability value. PNE 3 was formulated with 5% oil, 45% water, and 50% S_mix_, and possessed DS of 44.93 ± 0.60 nm (predicted 44.93 nm) and PDI of 0.17 ± 0.04 (predicted 0.17). PNE 3 showed a %T of 94.30 ± 0.00 (predicted 94%) and released 97.56 ± 1.51% (predicted 96.54%) of its drug content in 8 h.

### TEM of the optimized PNE

TEM micrograph of PNE 3 is displayed in Fig. 3II (left side), and it revealed a spherical droplet shape with narrow size distribution comparable to that obtained by the Zetasizer, Fig. 3II (right side), where no signs of aggregation or coalescence were observed.

### Preparation and characterization of PNE 3 gel

The gel base was prepared from HPMC and pluronic F127 at a ratio of 1:4 (w/w), then PNE 3 was mixed with the gel base at a ratio of 1:1. The viscosity of the prepared PNE 3 gel was 477 ± 43 cp, which demonstrates acceptable retention of the formulation on the site of application. In addition, the viscosity of the gel formulation declined with leveling up the shear rate, showing non-Newtonian flow (shear thinning), which is favorable with the topical application of dosage forms^57^. The therapeutic efficacy of gel formulation is directly linked to their spreadability and it plays an important role in patient compliance with the treatment. The spreadability of PNE 3 gel was calculated and was found to be 20.76 ± 2.21, signifying the ease of spreading of the formulation. The pH value recorded for PNE 3 gel was 4.86 ± 0.10 which probably would not produce skin irritation due to its nearness to the skin pH (pH 4.5–6.0)^58^.

### In vitro dissolution profile and stability studies of PNE 3 gel

The in vitro drug release profile of PNE 3 gel is illustrated in Fig. 3IB. It could be observed that incorporation of PNE 3 into a gel base resulted in sustaining the release of SAH for 24 h, which could be discussed in light of the increased viscosity that retarded the release of the drug. Regarding the results of the stability study, PNE 3 gel showed no signs of precipitation or change in color, in addition, there was a nonsignificant difference (*P* > 0.05) in the values of pH, and the viscosity between the stored samples at 40 °C ± 2 °C/75% RH ± 5% RH for 3 months and the freshly prepared ones.

### CLSM study

**Figure 4:**
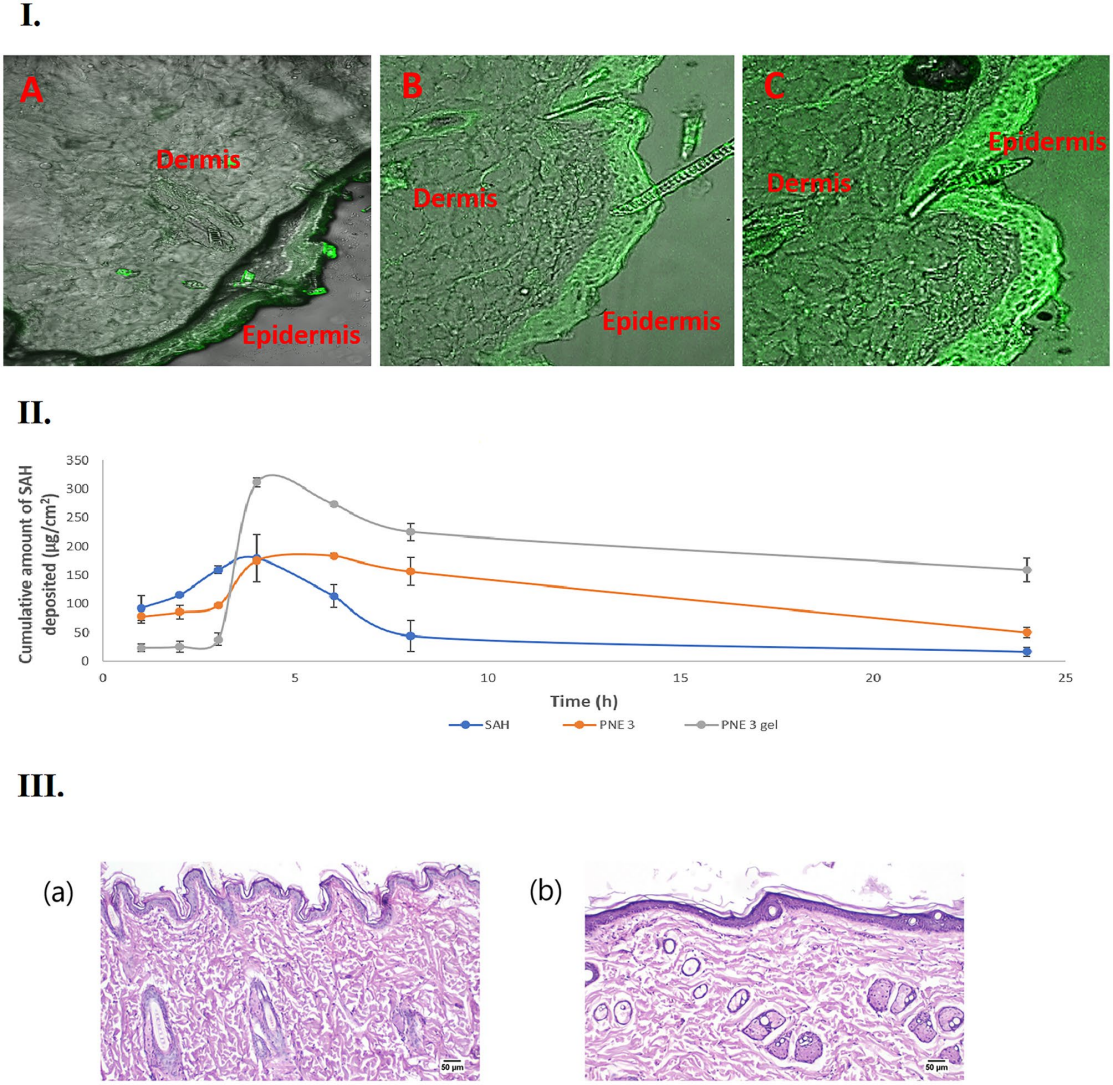
(**I**) Confocal laser scanning micrographs (CLSMs), obtained after 8 h following the application of Rhodamine B dye aqueous solution (**A**), Rhodamine B dye-loaded PNE 3 (**B**), and Rhodamine B dye-loaded PNE 3 gel (**C**), (**II**) The plot of the amount of SAH deposited per unit area in rat skin after the application of SAH, PNE 3, and PNE 3 gel, **and **(**III**) Histopathological photomicrographs of skin tolerance study; showing histopathological sections (hematoxylin- and eosin-stained) of (**a**) Untreated rat’s skin (group A); showing a normal histological structure of the epidermis and the skin appendages, and (**b**) Rat’s skin treated with SAH-loaded PNE 3 gel (group B) showing an apparent normal architecture of the epidermis and the dermis at 50 µm.

CLSM imaging affords information about the degree and pathways of the nanosystem's penetration through the skin. Figure 4I. exhibits the confocal images of the skin samples after the topical application of the dye solution (A), the dye-loaded PNE 3 (B), and the dye-loaded PNE 3 gel (C). The lowest intensity of the fluorescence was observed with SAH mainly in the epidermis layer, while a higher intensity of the fluorescence was detected at the epidermis and the deeper dermis layers with PNE 3. This could be attributed to the interrupted barrier via the increased fluidity of the skin lipids due to the effect of formulation components; namely the oil and the S_mix_ of the PNE^59,60^. However, it is observable that the fluorescence in the micrographs of skin treated with PNE 3 gel, compared to that of PNE 3, is highly diffused throughout the whole layers of the skin, principally as per the increased viscosity of the gel base, which allowed the retention of the formulation for a longer period of time at the site of application.

### Skin deposition study

The capability of a delivery system to shuttle a drug through the skin layers can be evaluated via the in vivo deposition study. Figure 4II. displays the plot of the amount of SAH deposited per unit area in rat skin after the application of SAH, PNE 3, and PNE 3 gel. It can be observed that PNE 3 gel was capable of depositing elevated amounts of SAH in the dermal tissue related to PNE 3 and SAH. The calculated AUC_0-24_ of PNE 3 gel (4398.98 ± 126.55 µg.h/cm^2^) was found to be pointedly higher (*P* < 0.05) than that of PNE 3 (2696.23 ± 201.74 µg.h/cm^2^) and that of SAH (1384.75 ± 54.53 µg.h/cm^2^), where PNE 3 gel was able to increase the AUC by 2.63 folds and 3.18 folds compared to PNE 3 and SAH, respectively. Thus, it could be said that PNE 3 gel enhanced the skin deposition of SAH upon topical application, via higher viscosity and retention potentialities of the gel base and improved penetration of the stratum corneum barrier because of the oil and S_mix_ content of PNE.

### Histopathological study

The histological micrographs of the rat skin of the control group (Group A) and the group treated with PNE 3 gel (Group B) are displayed in Fig. 4III. Group (A) showed normal skin histological structure of epidermis, dermis, and skin appendages. The skin of group (B) treated with PNE 3 gel did not display any significant changes in the microscopic structure of the skin, where the surface epithelium lining and the dermis structure of the skin were intact. The ultra-structure of skin morphology was not altered and the epithelial cells looked generally unaffected. In conclusion, the histopathological results confirmed the safety of the topical application of PNE 3 gel on the skin.

## Conclusion

The methanolic extract of *A. Heterophylla* was standardized against 4ʹʹʹmethoxyamentoflavone after being isolated and identified using ^1^H and ^13^C NMR. A validated UPLC-MS/MS method was developed for both standardization and further quantitative analysis of SAH. After standardization, it was found that 4ʹʹʹmethoxyamentoflavone is a major constituent, where one gram of SAH contained 20 mg of 4ʹʹʹmethoxyamentoflavone. The antibacterial and antifungal activity results revealed that the MIC of SAH was 0.156 mg/mL in the case of standard *S. aureus and E.coli,* however, SAH was not effective against the standard strain of *C.albicans*. A simplex lattice design was adopted for the optimization of PNEs. The optimized formula (PNE 3) showed minimal DS and PDI with maximum %T and extended-release profile for 8 h. PNE 3 was then loaded in a gel base (HPMC: Pluronic F-127, in ratio 1:4) which showed shear-thinning rheological behavior, acceptable pH, and a controlled release profile for 24 h. Moreover, PNE 3 gel exhibited enhanced skin penetration compared to PNE 3 and SAH via the CLSM study. The in vivo study confirmed the improved deposition of SAH after the application of PNE 3 gel compared to PNE 3 and SAH. Finally, the safety profile of PNE 3 gel was proved by the normal skin structure observed after histopathological examination. Consequently, it could be concluded that PNE 3 gel (loaded with 20% SAH) is a promising topical broad-spectrum antibacterial formulation for the treatment of skin infections.

## Data Availability

All data generated or analyzed during this study are included in this published article.
